# Oncogenic *RAS*-induced senescence in human primary thyrocytes: molecular effectors and inflammatory secretome involved

**DOI:** 10.18632/oncotarget.2013

**Published:** 2014-05-26

**Authors:** Maria Grazia Vizioli, Joana Santos, Silvana Pilotti, Mara Mazzoni, Maria Chiara Anania, Claudia Miranda, Sonia Pagliardini, Marco A. Pierotti, Jesus Gil, Angela Greco

**Affiliations:** ^1^ Molecular Mechanism Unit, Department of Experimental Oncology and Molecular Medicine, Fondazione IRCCS Istituto Nazionale dei Tumori, Milan, Italy; ^2^ Cell Proliferation Group, MRC Clinical Sciences Centre, Imperial College London, Hammersmith Campus, London, UK; ^3^ Laboratory of Molecular Pathology, Department of Pathology, IRCCS Foundation - Istituto Nazionale dei Tumori, Milan, Italy; ^4^ Scientific Directorate, IRCCS Foundation - Istituto Nazionale dei Tumori, Milan, Italy

**Keywords:** thyroid carcinoma, senescence, oncogenes, tumour suppressor, SASP

## Abstract

Oncogene-induced senescence (OIS) is a robust and sustained antiproliferative response to oncogenic stress and constitutes an efficient barrier to tumour progression. We have recently proposed that OIS may be involved in the pathogenesis of thyroid carcinoma by restraining tumour progression as well as the transition of well differentiated to more aggressive variants. Here, an OIS inducible model was established and used for dissecting the molecular mechanisms and players regulating senescence in human primary thyrocytes. We show that oncogenic *RAS* induces senescence in thyrocytes as judged by changes in cell morphology, activation of p16^INK4a^ and p53/p21^CIP1^ tumour suppressor pathways, senescence-associated β-galactosidase (SA-β-Gal) activity, and induction of proinflammatory components including IL-8 and its receptor CXCR2. Using RNA interference (RNAi) we demonstrate that p16^INK4a^ is necessary for the onset of senescence in primary thyrocytes as its depletion rescues *RAS*-induced senescence. Furthermore, we found that IL-8/CXCR2 network reinforces the growth arrest triggered by oncogenic *RAS*, as its abrogation is enough to resume proliferation. Importantly, we observed that CXCR2 expression coexists with OIS markers in thyroid tumour samples, suggesting that CXCR2 contributes to senescence, thus limiting thyroid tumour progression.

## INTRODUCTION

Thyroid carcinoma accounts for about 1% of all human cancers and its incidence has steadily increased over the last few decades. The majority of thyroid tumours arises from follicular thyroid cells and includes: papillary (PTC), follicular (FTC), poorly differentiated (PDTC) and anaplastic (ATC) carcinoma [1].

PTC is the most frequent thyroid cancer; it includes several histological variants characterized by different prognosis, from papillary thyroid microcarcinoma (PTMC) generally associated to an indolent course, to tall cell variant (TCV) associated to higher disease-related mortality and recurrence rates [2]. PTC is characterized by alternative driving genetic lesions converging on the RTK/RAS/BRAF/MAPK signaling pathway in about 70% of cases. Novel molecules and mechanisms relevant in PTC pathogenesis have been suggested by genes and miRNAs expression studies [3]. Nevertheless, the mechanisms underlying the onset and progression of this neoplasia have not been fully elucidated.

We have recently provided new insights into the molecular mechanisms of thyroid carcinogenesis by demonstrating, as first, the involvement of oncogene-induced senescence (OIS) [4].

OIS is a highly stable cell cycle arrest triggered by oncogenic stress in primary cells [5]. During OIS cells undergo multiple phenotypic changes, that include changes in morphology, metabolism, chromatin organization, an increase and reorganization in lysosome numbers (resulting in senescence-associated β-galactosidase (SA-β-Gal) activity), and the production of a complex secretome (referred as the SMS or SASP)[6;7]. Although there is debate on how best to define senescence there is an agreement that together with the stable cell cycle arrest several of the mentioned phenotypes (sometimes referred to as geroconversion) must occur, to distinguish senescence from cell cycle arrest [8].

One of the initial molecular triggers of OIS is the activation of a robust DNA-damage checkpoint response (DDR) caused by hyperproliferation and DNA hyper-replication resulting from oncogene expression [9;10]. Although DDR is not a universal feature of OIS [7;9;10] when present it contributes to activate p53-dependent reponses such as the upregulation of the cyclin-dependent kinase (CDK) inhibitor p21^CIP1^. The activation of the p53/p21^CIP1^ pathway, together with upregulation of p16^INK4a^ are the key to implement the senescent growth arrest [7]. The relative contribution of p16^INK4a^ and p53/p21^CIP1^ to senescence depends of the trigger and cell type where senescence occurs [11].

It is now appreciated that OIS represents an important barrier against tumour development *in vivo*: a number of studies have shown that premalignant lesions from human and mice are enriched in senescent cells. For instance, benign melanocytic nevi, which frequently carry the *BRAF^V600E^* oncogene, show features of senescence including SA-β-Gal activity and high p16^INK4a^ expression [12]. Moreover, senescence markers were found in human dermal neurofibromas, murine lung adenomas, human and murine prostatic adenomas, murine pancreatic intraductal neoplasias and murine lymphomas [13-18].

Notably, OIS is tightly connected with inflammation. Senescent cells display the “senescence associated secretory phenotype” (SASP): they produce a wide range of inflammatory cytokines and growth factors which operate in a cell-autonomous manner, but also they communicate with and modify the microenvironment [6;19;20]. Through the SASP senescent cells can affect their microenvironment in opposing ways and reinforce senescence or promote carcinogenesis. The SASP can induce senescence in normal or low-grade premalignant cells but also can boost cancer progression programs in high-grade premalignant or malignant cells [21]. In addition, SASP factors can trigger senescence surveillance, an immuno-mediated clearance of senescent cells, recently proposed as an important extrinsic component of the senescence anti-tumour barrier [22].

We have previously demonstrated that OIS may represent a barrier to thyroid epithelial tumour progression. The expression of PTC-associated oncogenes (*BRAF, RAS, RET/PTC* and *TRK*) triggers senescence in human primary thyrocytes, as demonstrated by lack of proliferation, changes in cell morphology, induction of various OIS specific biomarkers including elevated SA-β-Gal activity, presence of senescence-associated heterochromatic foci (SAHF), as well as increased expression of p16^INK4a^, p21^CIP1^ and p53, and robust ERK1/2 activation. Furthermore, immunohistochemical analysis of a panel of thyroid tumours characterized by increasing aggressiveness showed that expression of OIS markers is upregulated at early tumour stages, and lost at more advanced ones, suggesting that OIS may influence the pathogenesis of thyroid carcinoma by constraining neoplastic transformation [4]. Of note, thyrocytes undergoing OIS model *in vitro* the early thyroid tumour stages.

Here we established an inducible system of OIS in thyrocytes. OIS could be induced in thyrocytes by adding 4-hydroxytamoxifen (4OHT) which induce the expression of an ER:RAS fusion protein. Using this system, we show that p16^INK4a^ is a regulator of *RAS*-mediated senescence. We also report that upon *RAS* activation thyrocytes show increased levels of several proinflammatory components including IL-8 and its receptor CXCR2 that act in reinforcing cell cycle arrest *in vitro*. Importantly, we also observed that CXCR2 expression coexists with senescence markers in thyroid tumour samples, in line with the view that senescence is linked with inflammation.

## RESULTS

### Establishment and characterization of *ER:RAS* inducible system in thyrocytes

**Figure 1:**
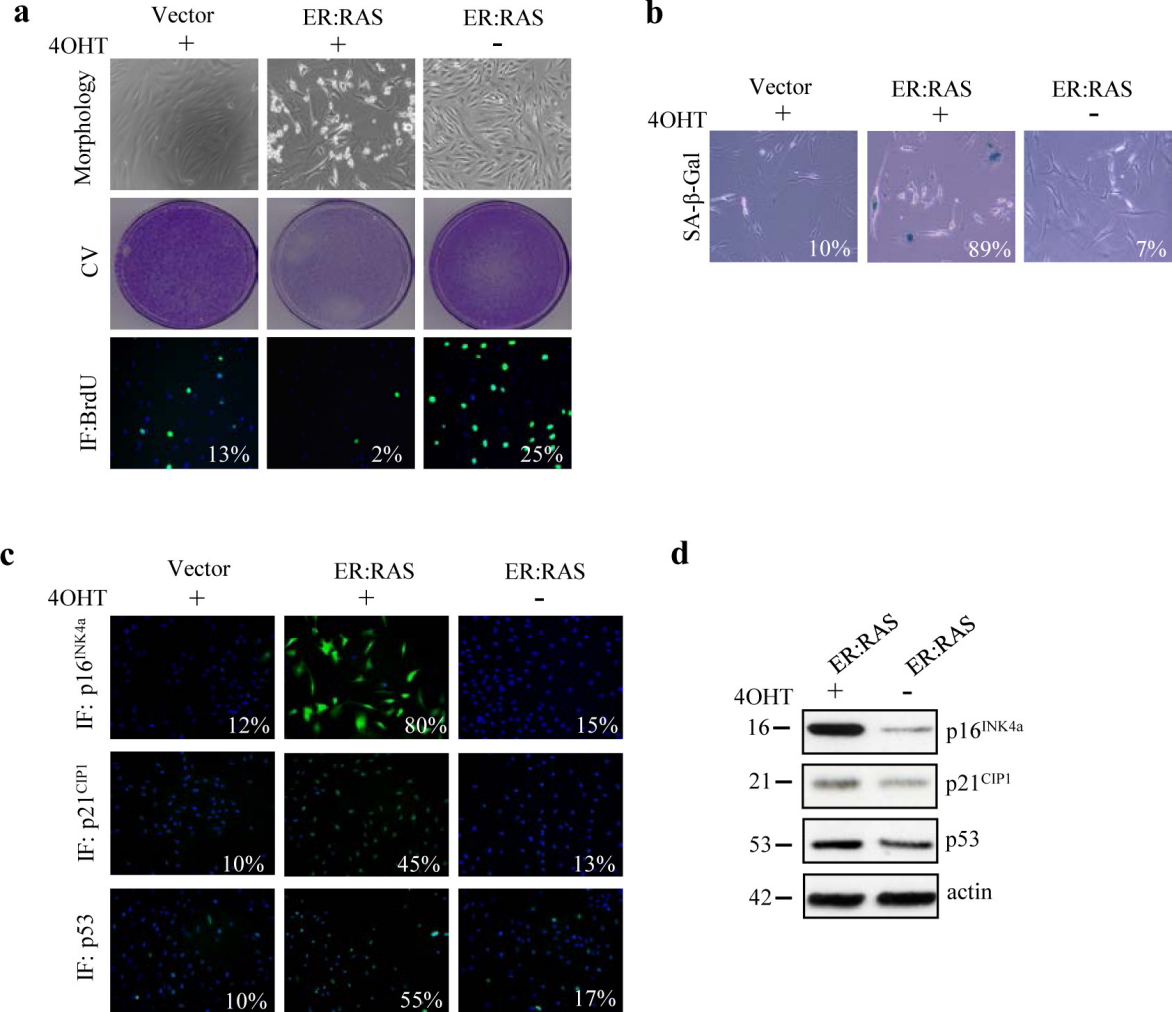
Oncogenic *RAS* triggers senescence in human primary thyrocytes Cells carrying empty vector (Vector) or *ER:RAS* were left untreated or treated with 4OHT for 7 days. (a) Representative pictures showing cell morphology (upper panel, magnification 10x), Crystal violet staining (CV, middle panel) and BrdU incorporation detected by immunofluorescence (IF, bottom panel, magnification 10x). (b) SA-β-Gal staining (magnification 20x). (c) Expression of p16^INK4a^ (upper panel), p21^CIP1^ (middle panel) and p53 (bottom panel) determined by IF; magnification 10x. The percentage of positive cells is indicated in each photograph. High Content Analysis (HCA) was used for BrdU, p16^INK4a^, p21^CIP1^ and p53 quantification. For SA-β-Gal staining at least 200 cells were counted. (d) Determination by western blotting of protein levels as indicated in an independent experiment; β-actin serves as loading control. The molecular weight of each protein is indicated. Data are from a representative out of two independent experiments.

Our previous studies showed that PTC-associated oncogenes, including *H-RAS^G12V^*, trigger senescence in human primary thyrocytes [4]. Even if *BRAF^V600E^* is the most common mutation in PTC, *H-RAS^G12V^* is highly prevalent in PTC with follicular variant histology [23]. Thus, to better understand the molecular mechanisms and the players required for the induction and maintenance of senescence in our cellular setting we took advantage of the well established *ER:RAS* inducible retroviral vector carrying *H-RAS^G12V^* oncogene fused to a 4-hydroxytamoxifen (4OHT)-responsive Estrogen Receptor ligand binding domain [24]. Primary thyrocytes were transduced with *ER:RAS* retroviral vector or empty vector, and two days later were selected with Geneticin (G418) for approximately 15 days. Upon 7 days of 4OHT treatment, we monitored the effects of *RAS* expression on primary thyrocytes by analyzing several senescence markers. 4OHT-treated *ER:RAS* cells displayed changes in morphology, becoming flat and enlarged. No morphology changes were detected in the untreated counterparts or in the cells transduced with the empty vector (Figure 1a, top panel). Moreover, activation of *RAS* induced growth arrest, whereas controls continued proliferating. For instance, as evident by crystal violet staining we observed a decrease in cellular density in 4OHT-treated *ER:RAS* thyrocytes compared with untreated *ER:RAS* and empty vector (Figure 1a, middle panel). This was also corroborated with a BrdU incorporation assay: 4OHT-treated *ER:RAS* thyrocytes exhibited a stark reduction of S phase (2% of cells incorporated BrdU) when compared with the controls (25% and 13% of untreated and empty vector cells, respectively, incorporated BrdU) (Figure 1a, lower panel). To further prove that the growth arrest had characteristics of senescence, cells were stained for SA-β-Gal activity. Following *RAS* activation, a higher percentage (89%) of cells displayed SA-β-Gal activity compared with the percentage of positive cells in the untreated *ER:RAS* or empty vector (7% and 10% respectively) (Figure 1b). To address the mechanisms by which induction of *RAS* triggers senescence in primary thyrocytes, we analyzed by immunofluorescence the expression of some well-known senescence effectors. After 4OHT treatment *RAS* caused a marked increase in the expression of p16^INK4a^ (80% vs 15% and 12% of control cells), p21^CIP1^ (45% vs 13% and 10% of control cells) and p53 (55% vs 17% and 10%) (Figure 1c). The upregulation of p16^INK4a^, p21^CIP1^ and p53 in senescent thyrocytes was also confirmed by western blot analysis (Figure 1d). Taking these results together we conclude that *ER:RAS* thyrocytes undergo senescence upon *RAS* activation, and thus they represent a powerful tool to get more insights into the molecular mechanisms and players involved in OIS thyrocytes.

### The relative contribution of p16^INK4a^, p53 and p21^CIP1^ in *RAS*-induced senescence in thyrocytes

**Figure 2:**
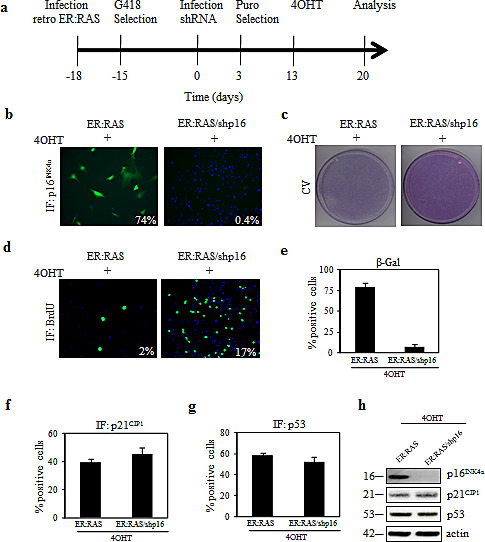
(a) Timeline of the experiments in this Figure and in Figures 3 and 6 *ER:RAS* cells retrovirally transduced with the indicated shRNA, and selected with puromycin, were treated with 4OHT for 7 days and then analyzed. (b-g) Knockdown of p16^INK4a^ alleviates *RAS*-induced senescence in primary thyrocytes. *ER:RAS* cells with intact or null-p16^INK4a^ were analyzed for: p16^INK4a^ expression (b), proliferative potential through CV (c) and BrdU incorporation (d), SA-β-Gal activity (e), and expression of p21^CIP1^ (f) and p53 (g). High Content Analysis (HCA) was used to quantify the IF staining against p16^INK4a^, BrdU, p21^CIP1^ and p53. The percentage of SA-β-Gal positive cells was determined by counting at least 200 cells. (h) Determination by western blotting of protein levels as indicated, in an independent experiment; β-actin was used as loading control; the molecular weight of each protein is indicated. Images from a representative out of two independent experiments are shown; in the charts the mean ± s.d. of two independent experiments is reported. shp16: short hairpin targeting p16^INK4a^; CV: crystal violet; IF: immunofluorescence.

To examine the requirement of p16^INK4a^ or p53 to *RAS*-mediated thyrocytes senescence we applied gene silencing with shRNA delivered by retroviral vector. *ER:RAS* cells were transduced with shRNA targeting p16^INK4a^ (shp16) or p53 (shp53) and selected for the integration of the construct with puromycin for approximately 10 days. Afterwards, cells were treated with 4OHT and analysed 7 days later (Figure 2a).

Regarding p16^INK4a^ we observed a strong suppression of protein expression in shp16 transduced cells as judged by immunofluorescence (Figure 2b) and western blot analysis (Figure 2h). p16^INK4a^ knockdown caused 4OHT-treated *ER:RAS* cells to recover proliferation capability, as evident by crystal violet staining (Figure 2c). To quantify such rescue we performed a BrdU incorporation assay (Figure 2d): the percentage of 4OHT-treated *ER:RAS* cells incorporating BrdU increased upon knockdown of p16^INK4a^ and reached 17% compared with 2% of p16^INK4a^-intact *ER:RAS* cells. Similar results were also observed following transfection of p16^INK4a^ siRNA or control siRNA (scramble) into *ER:RAS* thyrocytes (data not shown). Moreover, in shp16 transduced cells the ability to restart proliferation was accompanied by a clear reduction of SA-β-Gal activity: in fact only 5% of cells positively stained for SA-β-Gal versus 80% of p16^INK4a^ -intact *ER:RAS* cells (Figure 2e). The depletion of p16^INK4a^ did not affect the level of p21^CIP1^ and p53 proteins, as shown by immunofluorescence (Figure 2f-g) and western blot (Figure 2h). Hence, we conclude that disruption of p16^INK4a^ allows primary thyrocytes to escape from *RAS*-induced senescence.

**Figure 3:**
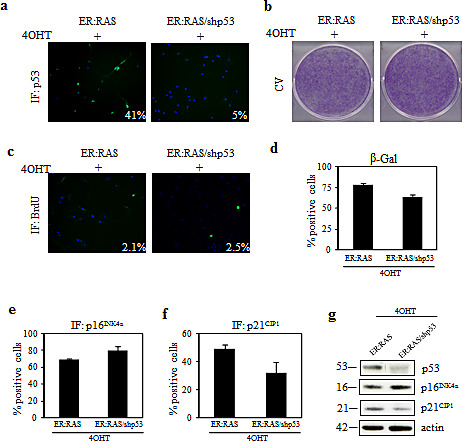
Effect of p53 knockdown on *RAS*-induced senescence in primary thyrocytes (a-e) *ER:RAS* cells with intact or null p53 were analyzed for: p53 expression (a), proliferative potential through CV (b) and BrdU incorporation (c), SA-β-Gal activity (d), expression of p16^INK4a^ (e) and p21^CIP1^ (f). High Content Analysis (HCA) was used to quantify IF for p53, BrdU, p16^INK4a^ and p21^CIP1^. Data are from a representative out of two independents experiments. Error bars represent the standard deviation. The percentage of positive cells for SA-β-Gal staining is obtained by counting at least 200 cells. (g) Western blotting analysis for the expression of the indicated proteins performed in an independent experiment; β-actin was used as loading control; the molecular weight of each protein is shown. shp53: short hairpin targeting p53; CV: crystal violet ; IF: immunofluorescence.

With respect to p53, an efficient knockdown was obtained by shp53 transduction: following 4OHT treatment only 5% of p53-null *ER:RAS* thyrocytes displayed p53 expression compared with 41% of p53-intact *ER:RAS* cells, as assessed by immunofluorescence (Figure 3a). Such depletion was also confirmed by western blot analysis (Figure 3g). However, in response to 4OHT treatment, no difference in cell proliferation was observed between p53-intact and p53-null *ER:RAS* thyrocytes, as evident by crystal violet staining (Figure 3b) and BrdU incorporation (Figure 3c). Similar results were also obtained after p53 siRNA transfection. We again observed that depletion of p53 had no effect on thyrocytes growth inhibition induced by *RAS* activation (data not shown). Likewise, upon p53 depletion we did not observe a significant difference in SA-β-Gal activity, as well as in p16^INK4a^ expression. In particular, in 4OHT-treated *ER:RAS* thyrocytes, SA-β-Gal activity was detected in 65% of p53-null and in 77% of p53-positive cells (Figure 3d). Similarly, the induction of p16^INK4a^ was maintained in p53-null cells (69% vs 80% of control) (Figure 3e), whereas a slight decrease of p21^CIP1^ was detected in p53-null cells (Figure 3f). The same trend was also observed by western blot analysis, as shown in Figure 3g.

**Figure 4:**
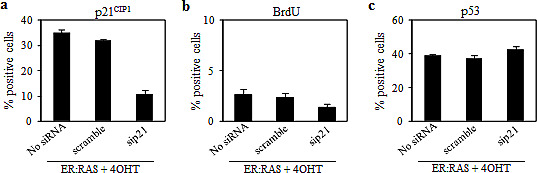
Effect of p21^CIP1^ depletion on *RAS*-induced senescence in primary thyrocytes (a-c) Untrasfected *ER:RAS* cells (No siRNA) and *ER:RAS* cells transfected with a siRNA control (scramble) or with a siRNA targeting p21^CIP1^ (sip21) treated with 4OHT for 4 days, were subjected to IF for: p21^CIP1^ expression (a), BrdU incorporation (b), and p53 expression (c). High Content Analysis (HCA) is used for the quantification. Data are the mean ± s.d. of two independent experiments.

To investigate the role of p21^CIP1^ in mediating OIS, *ER:RAS* thyrocytes were reverse-transfected with siRNA targeting p21^CIP1^. After 24 hours cells were treated with 4OHT and analysed 4 days later (i.e.: 5 days post silencing). Cells untrasfected or transfected with scrambled siRNA served as negative controls. As shown in Figure 4a, an efficient silencing of p21^CIP1^ was observed. Such depletion did not influence the growth arrest induced by activation of *RAS*: only 2% of 4OHT-treated p21^CIP1^-null *ER:RAS* cells resulted positive for BrdU incorporation, an amount similar to the controls (Figure 4b). No effect of p21^CIP1^ silencing on p53 expression was observed: after 4OHT treatment, in p21^CIP1^-null *ER:RAS* cells the levels of p53 remained high and comparable to the ones observed in the control cells (Figure 4c).

Together our data clearly demonstrate that p16^INK4a^ is an essential effector of senescence in thyrocytes.

### SASP components increase during *RAS*-induced senescence in thyrocytes

**Figure 5:**
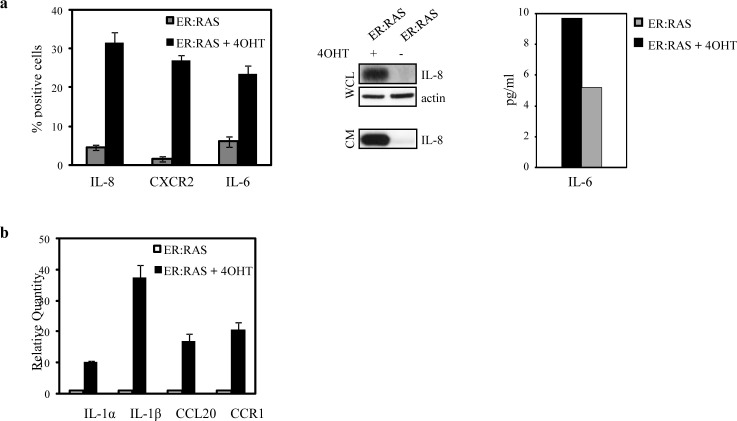
Upregulation of proinflammatory components during senescence in primary thyrocytes *ER:RAS* cells were treated or not with 4OHT for 7 days. (a) IL-8, CXCR2 and IL-6 expression assessed by IF. High Content Analysis (HCA) is used for the quantification. Data are the mean ± s.d. of two independent experiments (left panel); determination of IL-8 protein level in whole cell lysates (WCL) and conditioned medium (CM) by western blotting (middle panel); determination of IL-6 levels in CM by ELISA (right panel). (b) qRT-PCR analysis of IL-1α, IL-1β, CCL20 and CCR1 transcript levels normalized with HPRT housekeeping gene and expressed respect to the control.

In addition to classical senescence players like p16^INK4a^, p21^CIP1^ and p53, a complex cocktail of factors including proinflammatory cytokines, chemokines, extracellular proteases, matrix components and growth factors, regulates the initiation and maintenance of senescence [25-28]. We investigated the expression of some SASP factors and cognate receptors. IL-8 and IL-6 are considered two of the most important cytokines secreted by senescent cells [25;26]. We analyzed the protein expression of IL-8, its receptor CXCR2, and of IL-6 via immunofluorescence. *ER:RAS* cells responded to *RAS* activation with high expression of IL-8/CXCR2 and IL-6, whereas the levels detected in the untreated counterparts were very low (Figure 5a, left panel). We also found that both cytokines are present in the conditioned medium of senescent thyrocytes, as shown by western blot analysis for IL-8 and ELISA for IL-6 (Fig.5a, middle and right panels).

We also quantified the mRNA levels of IL-1α, IL-1β, the CC chemokine CCL20, and the CC chemokine receptor CCR1 by quantitative real time PCR (qRT-PCR). As shown in Fig. 5b, transcripts encoding IL-1α and IL-1β, CCL20 and CCR1 increased when thyrocytes underwent senescent with respect to untreated *ER:RAS* cells. These results indicate that, as in others cellular models, a SASP-like response is activated in senescent thyrocytes.

### A critical role for IL-8/CXCR2 axis in mediating *RAS*-induced senescence in thyrocytes

To better understand the connection between senescence and inflammation in our cellular model we focused on IL-8. Notably, IL-8 is a well-known mediator of senescence in normal cells, and it has also been suggested as tumour progression factor in thyroid cancer, through increasing angiogenesis and vascularisation, metastatic spread and poor prognosis [29-31].

**Figure 6:**
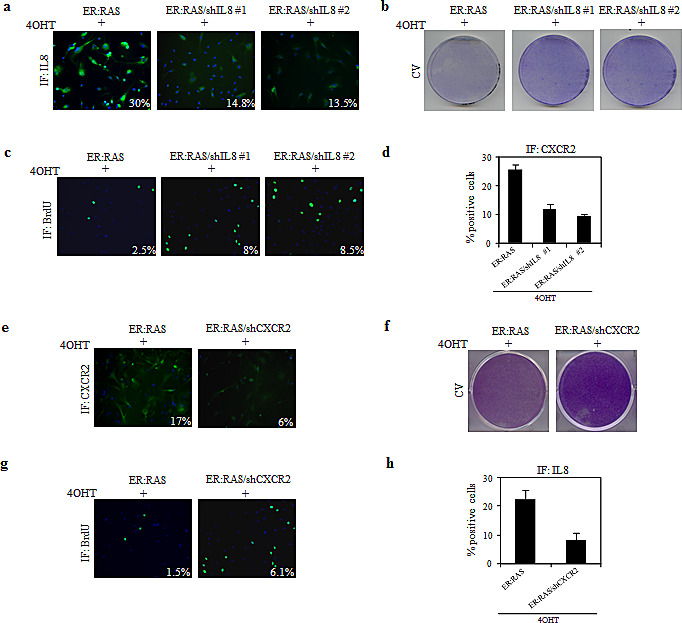
Role of IL-8/CXCR2 axis in mediating OIS in primary thyrocytes (a-d) *ER:RAS* cells with intact or null IL-8(shIL8) were analyzed for: IL-8 expression (a), proliferative potential through CV (b) and BrdU incorporation (c), and CXCR2 expression (d). The levels of IL-8, BrdU and CXCR2 were analyzed by IF and quantified by High Content Analysis (HCA). (e-h) *ER:RAS* cells with intact or null CXCR2 (shCXCR2) were analyzed for: CXCR2 expression (e), proliferative potential through CV (f) and BrdU incorporation (g), and IL-8 expression (h). CXCR2, BrdU and IL-8 immunofluorescence was quantified by High Content Analysis (HCA). Images from a representative out of two independent experiments are shown; in the charts the mean ± s.d. of two independent experiments is reported. shIL8#1 and 2: short hairpins targeting IL8; shCXCR2: short hairpin targeting CXCR2; CV: cristal violet; IF: immunofluorescence.

To investigate the IL-8 involvement in thyrocyte senescence we knocked down its expression with two independent retroviral shRNAs (#1, #2) which reduced by approximately 50% the number of IL-8 positive cells (Figure 6a). Such IL-8 suppression was sufficient to restore the proliferation capability in 4OHT-treated *ER:RAS* cells, as shown by crystal violet staining and BrdU incorporation (Figure 6b-c). For instance, the percentage of 4OHT-treated *ER:RAS* cells actively replicating DNA in the absence of IL-8 was 8% and 8.5% (shIL-8 #1 and #2, respectively) versus 3% of control cells (Figure 6c). Interestingly, upon IL-8 abrogation CXCR2 positive cells were 30% in IL-8 intact cells, and 13% and 9% in IL-8 knocked down cells (Figure 6d), suggesting that IL-8 suppression confers growth advantage by reducing CXCR2 expression.

In keeping with the well-known connection between IL-8 and its receptor CXCR2 for the induction and maintenance of OIS [26] and having shown that CXCR2 expression increased during senescence in our cellular model, we wondered whether CXCR2 is also required for the mediation of OIS. To this end, we used shRNA retroviral vector, which reduced CXCR2 protein level of approximately 60% (Figure 6e). This suppression led 4OHT-trated *ER:RAS* cells to resume proliferation, as demonstrated by crystal violet staining (Figure 6f). Such rescue was quantified by BrdU incorporation assay: following activation of *RAS* the percentage of positive cells increased by ~4-fold upon CXCR2 knockdown (Figure 6g). Depletion of CXCR2 caused a decrease in IL-8 availability: upon 4OHT treatment 9% of CXCR2-null *ER:RAS* cells were positive for IL-8 staining compared with 25% of the CXCR2-intact cells (Figure 6h). These results suggest that IL-8/CXCR2 axis plays a role in mediating OIS in *ER:RAS* transduced thyrocytes, being involved in growth arrest triggered by oncogenic stress.

### CXCR2 expression coexists with OIS markers in thyroid tumour samples

**Figure 7:**
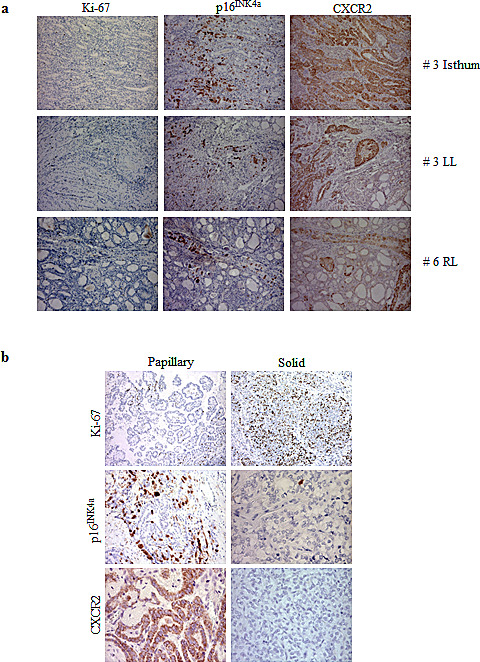
Immunohistochemical analysis for the expression of Ki-67, p16^INK4a^ and CXCR2 (a) Representative pictures of PMTC #3 and #6. LL: left lobe; RL: right lobe. (b) Papillary and solid areas of PDTC #13.

We have recently shown that signs of OIS, such as p16^INK4a^ upregulation and very low proliferation index are observed in PTMC, the early thyroid tumour stage, and then lost during tumour progression [4]. To investigate the trend of CXCR2 expression with respect to other OIS markers, a panel of 13 thyroid tumours was analyzed for the expression of p16^INK4a^, CXCR2 and Ki-67 by immunohistochemistry. As detailed in Table 1, tumour collection included 9 PTMCs (among which 3 bifocal and 1 trifocal) and 4 PTCs (3 not otherways specified (NOS) and one PDTC, the latter displaying both papillary and solid areas). All the PTMC samples showed a very low expression of the Ki-67 proliferation marker. Nine samples were scored positive for both CXCR2 and p16^INK4a^ expression, and in most cases stretches of p16^INK4a^ positivity largely overlapped with CXCR2 positive areas. Representative pictures from three samples are shown in Figure 7a. In the remaining 5 PTMC samples, focal CXCR2 positivity was also observed in tumour areas that were negative for p16^INK4a^ staining.

**Table 1:** Immunohistochemical analysis for p16^INKa^, CXCR2 and Ki-67 in thyroid carcinomas

	p16^INK4a^	CXCR2	Ki-67
**PTMC**			
1_2_ Isthum	+	+	vL
RL	+	+	vL
2_13_	+	+	vL
3 Isthum	+	+	vL
LL	+	+	vL
4	+	+	vL
5	-	+	vL
6 RL	+	+	vL
LL	+	+	vL
7	-	+	vL
8	+	+	vL
9 RL1	-	+	vL
RL2	-	+	vL
LL	-	+	vL
**PTC**			
*NOS*			
10_7_	+	+	L
11_4_	-	-	L
12	-	-	L
*PDTC*			
13_10_ papillary	+	+	L
solid	-	-	H

PTMC: papillary thyroid microcarcinoma; PTC: papillary thyroid carcinoma; NOS: not otherways specified; PDTC: poorly differentiated thyroid carcinoma; RL: right lobe; LL: left lobe. p16^INK4a^ and CXCR2 immunostaining was scored positive (+) when focal tumour areas with at least 10% of immunolabeled cells were present. Ki-67 staining was scored as very low (vL, 1−5% positive cells), low (L, 6−10% positive cells), medium (M, 11−30% positive cells) and high (H, ≥ 31% positive cells). Subscript numbers indicate the corresponding case number reported in a previous paper [4].

The three PTC NOS samples displayed low proliferation index. One sample expressing p16^INK4a^ was also positive for CXCR2, whereas the others two samples were scored negative for both p16^INK4a^ and CXCR2.

The co-existence of OIS markers and CXCR2 is strengthened by the analysis of a PDTC sample (#13), representing a model of in situ tumour progression: it displays areas with papillary and solid pattern of growth, previously shown, positive and negative for the expression of senescence markers [4]. As shown in Table 1 and Figure 7b the papillary areas, associated with low proliferation index, were positive for both p16^INK4a^ and CXCR2, whereas the solid areas, with high Ki-67 expression, were negative. This finding corroborates the notion that loss of CXCR2 contributes to escape senescence.

## DISCUSSION

We have recently proposed that OIS, a mechanism recognized as a barrier against tumor development, may be involved in the pathogenesis of thyroid carcinoma. In particular, we have reported that PTC-associated oncogenes, including *H-RAS^G12V^*, induce senescence in human primary thyrocytes [4]. As tool for a deeper analysis of OIS thyrocytes, here we established and characterized an inducible model of thyrocytes senescence based on *ER:RAS* inducible system. Activation of *RAS* led to senescence-associated characteristics, as judged by the presence of growth arrest, changes in cell morphology, increased SA-β-Gal activity and upregulation of p16^INK4a^, p21^CIP1^ and p53.

As reported in several studies the critical role of p53 and p16^INK4a^ in senescence is not universal across cells type and it differs between mouse and human cells. For instance, in mouse embryo fibroblasts disruption of p53 is sufficient to prevent senescence [32]. Likewise, inactivation of pRb gene together with other members of Rb family such as p107 and p130, but not pRb alone, is sufficient to escape senescence [33]. Unlike the behaviour of mouse cells, the inactivation of both p53 and pRb is essential to prevent the onset of replicative senescence in human cells [34], whereas disruption of only one of these proteins delays the onset of senescence.

To assess the relative contribution of the p16^INK4a^, p53 and p21^CIP1^ in the thyrocytes senescence program we used RNA interfering strategy. In particular, in our experimental setting, depletion of p53 had no impact on senescence upon oncogenic stress. For instance, no change in cell proliferation, BrdU incorporation or SA-β-Gal activity was observed between p53-intact and p53-null senescent thyrocytes. Interestingly, p53 depletion reduced the levels of p21^CIP1^, whereas the expression of p16^INK4a^ was maintained. Similarly, p21^CIP1^ knockdown had no influence in the inhibition of proliferation induced by activation of *RAS*. On the contrary, the silencing of p16^INK4a^ was sufficient to abrogate the onset of senescence. This was illustrated by increased proliferative potential as well as decreased in SA-β-Gal activity. Thus, in our experimental setting p16^INK4a^ seems to be a relevant effector, supporting a model in which oncogenic *RAS* acts primarily through the p16^INK4a^ pathway in thyrocytes.

It is important to note that these results are in contrast with our previous findings in *BRAF^V600E^* lentivirally transduced thyrocytes, where the inactivation of p16^INK4a^ was not sufficient to prevent induction of senescence, suggesting that the critical role of p16^INK4a^ in the abrogation of senescence may also depend on different genetic context, as previously proposed by others [5;12]. However, in thyrocytes infected with retroviral vector encoding *BRAF^V600E^*, p16^INK4a^ silencing was able to overcome cell cycle arrest (unpublished results). Thus, the different oncogene expression levels due to the different methods of oncogene delivery may warrant the different response upon p16^INK4a^ depletion.

In the last few years, growing body of evidence suggests that OIS is connected with inflammation. Cells undergoing senescence produce increased amount of inflammatory cytokines and chemokines that play an essential role in the initiation and maintenance of cellular senescence [25;26].

We found that several proinflammatory components, including IL-8/CXCR2, IL-6, IL-1, as well as CCL20 and CCR2 are upregulated in *ER:RAS* senescent thyrocytes, thus suggesting that a SASP-like phenomenon may occurr.

Our results suggest that IL-8/CXCR2 axis plays a role in mediating thyrocytes OIS, being involved in growth arrest triggered by oncogenic stress. Knockdown of IL-8 using two independent shRNAs vectors allowed 4OHT-treated *ER:RAS* cells to circumvent cell cycle arrest and resulted in decreased CXCR2 levels. Similarly, upon depletion of CXCR2 by shRNA vector, 4OHT-treated *ER:RAS* thyrocytes were able to resume proliferation. We also observed a drop of IL-8 expression after CXCR2 abrogation, suggesting a coordinated regulation of IL-8 and its receptor during senescence.

In parallel to these observations *in vitro*, we observed a correlation between increased CXCR2 expression and OIS markers immunopositivity in human thyroid samples, suggesting that IL-8/ CXCR2 axis limits thyroid tumour progression. These findings are in harmony with previously models [25;26] in which the activation of the inflammatory network plays an essential role in OIS *in vitro* and *in vivo*.

On the other hand, it is well known that cytokines and chemokines have also been implicated in promoting cancer, increasing angiogenesis, vascularisation and metastasis [35].

A link between thyroid cancer and inflammation has also been recognized; thyroid carcinomas are associated with the constitutive expression of several proinflammatory cytokines (IL-8, IL-6, GROα, IL-1α, G-CSF, GM-CSF) that can be able to maintain the invasive phenotype [36;37]. It has been shown that all PTC-associated oncogenes (*BRAF, RAS* and *RET/PTC*) induces the up-regulation of cytokines and chemokines in normal thyroid cells; these factors can promote proliferation, invasion and metastasis [30;38]. Interestingly, in this study we have shown that in senescent thyrocytes IL-8 acts in concert with its receptor CXCR2 to reinforce senescence. What switches the secretome components from prosenescence to protumorigenic activity is not well understood: stage and status of lesion and its genetic background could be crucial factors. To note, CXCR2 mutation or downregulation cancels its prosenescent action facilitating tumour progression [26]. Moreover, several studies have yielded a wealth of evidence that some of these secreted proteins reinforce senescence by acting in autocrine manner, whereas they promote malignant phenotype by paracrine activity. Interestingly, Acosta et al. [39] recently demonstrated that these factors can trigger senescence by paracrine fashion *in vitro* and *in vivo* models.

In this paper we have used an inducible model for the characterization of OIS in thyrocytes. Such model, which mimics *in vitro* thyroid tumour early stages, represents a useful tool for a further dissection of the mechanisms governing OIS in thyrocytes and the interplay between senescent thyrocytes and microenvironment. This kind of studies, coupled to analyses performed in thyroid tumour samples characterized by different aggressiveness, as well as in corresponding cell lines, will allow defining the role of OIS players, including chemokines and cytokines, at early and late tumour stages. This will assess whether prosenescence therapy may represent a strategy for thyroid cancer treatment.

## METHODS

### Cell culture and treatments

Normal thyroid samples were obtained from patients undergoing surgery at IRCCS Istituto Nazionale dei Tumori (Milan, Italy). All patients gave their written informed consent, and the study was approved by the Independent Ethical Committee of Fondazione IRCCS Istituto Nazionale dei Tumori. Primary thyrocyte cultures were established and maintained in nutrient mixture Ham's F12 medium (custom made by Invitrogen, Carlsbad, CA, USA) containing 5% calf serum and bovine hypothalamus and pituitary extracts, as previously described [40].

4-hydroxytamoxifen (4OHT) (Sigma Aldrich, St Louis, Mo, USA) was used at 200 nM to activate *RAS* oncogene. Geneticin (G418, GIBCO, Carlsbad, CA, USA) and puromycin (Invitrogen, Carlsbad, CA, USA) were used for cell selection at 400 μg/ml and 2 μg/ml, respectively.

### Constructs and siRNA

Vector encoding *H-RAS^G12V^* has been described previously [41]. Knockdown of p16^INK4a^, p53, IL-8 and CXCR2 was achieved using validated shRNA retroviral vectors [26;41]. Silencing of p16^INK4a^, p53 and p21^CIP1^ was achieved using commercial siRNA (QIAGEN, Hilden, Germany), as described previously [26;41].

### Retrovirus production and infection

Methods used for retrovirus production and infection have been previously described [41].

### shRNA and siRNA transfection

For shRNA transduction *ER:RAS* cells were transduced with shRNA retroviral vector and maintained under puromycin selection for 10 days.

For siRNA transfection, *ER:RAS* cells were reverse-transfected with 30 nM siRNA using a 3.5% solution of Hiperfect (QIAGEN, Hilden, Germany). The Cy3-labeled siGLO cyclophilin B siRNA (Dharmacon, Buckinghamshire, UK) was used to monitor transfection efficiency. AllStars scrambled siRNA were used as negative controls.

### Cristal violet staining

Cells (1x10^5^) were plated in 10-cm dish. After 7 days cells were washed with PBS, fixed with 0.5% glutaraldehyde (w/v) in PBS for 30 minutes, and then stained with 0.2% crystal violet (w/v).

### BrdU (5-Bromo-2'deoxyuridine) incorporation assay

Cells were plated in 96-well plates and subsequently incubated with 5-Bromo-2' deoxyuridine (BrdU, 50 μM, Sigma Aldrich, St Louis, Mo, USA) for 24 hours. Cells were then washed with PBS, fixed for 30 minutes with 4% (w/v) paraformaldehyde (Sigma Aldrich, St Louis, Mo, USA), and subjected to immunofluorescence staining as previously described [26]. Briefly, after permeabilization for 5 minutes with 0.2 (v/v) Triton X-100 in PBS, and incubation for 30 minutes with 1X blocking solution (0.5% (w/v) BSA, 0.2% (w/v) fish skin gelatin), cells were incubated with anti-BrdU primary antibody (1:500, BD Becton Dickinson, NJ, USA) in the presence of DNase I (0.5U/μL; Sigma Aldrich, St Louis, Mo, USA) and 1 mM MgCl_2_ for 30 minutes. After washing with PBS cells were incubated with Alexa Fluor® 488 mouse secondary antibody (1:500, Invitrogen, Carlsbad, CA, USA) for 30 minutes and then incubated with DAPI (1.5 μM, Invitrogen, Carlsbad, CA, USA) for 30 minutes. Plates were examined using In Cell Analyser and High Content Analysis was performed to discriminate positive BrdU nuclei and total nuclei (IN Cell Analyser 1000, GE Healthcare Buckinghamshire, UK).

### Senescence-associated β-galactosidase (SA-β-Gal) assay

SA-β-Gal staining was performed as described previously [4]. Images were taken using the Olympus CKX41 inverted fluorescence microscope, supplied with a DP20 digital camera. The percentage of SA-β-gal positive cells were determined upon counting of at least 200 cells.

### Quantitative RT-PCR

RNA was isolated with TRIzol reagent (Invitrogen, Carlsbad, CA, USA) and purified with RNeasy purification kit (QIAGEN, Hilden, Germany). One μg of RNA was retrotranscribed using SuperscriptIII (Invitrogen, Carlsbad, CA, USA) following the manufacturer's instructions. For each sample, 20 ng of retro-transcribed RNA were amplified by PCR carried out in triplicate on an ABI PRISM 7900 using the following TaqMan gene expression assays: Hs00174092_m1 for IL-1α expression; Hs00174097_m1 for IL-1β expression; Hs00171125_m1 for CCL20 and Hs00174298_m1 for CCR1 expression. Human HPRT (HPRT-Hs99999909_m1) was used as housekeeping gene for normalisation. Data analysis was performed by the Sequence Detection System (SDS) 2.2.2 software (Applied Biosystems, Foster City, CA, USA).

### Immunofluorescence and high content analysis

Immunofluorescence staining was performed as for BrdU assay using the following primary antibodies: anti-p16^INK4a^ (JC8, 1:100, Santa Cruz Biotechnology Inc, Santa Cruz, CA, USA); anti-p21^CIP1^ (P1484, 1:100, Sigma Aldrich, St Louis, Mo, USA); anti-p53 (DO-1, 1:100, Santa Cruz Biotechnolgy Inc, Santa Cruz, CA, USA), anti-IL-8 (1:100, BD, Becton Dickinson, NJ, USA), anti-CXCR2 (1:100, BD, Becton Dickinson, NJ, USA), anti-IL-6 (1:100, BD Becton Dickinson, NJ, USA). Anti-mouse Alexa488-conjugated antibody (1:500, Invitrogen, Carlsbad, CA, USA) was used as secondary antibody. Acquisition of immunofluorescence images was performed using the IN Cell Analyzer 1000 automated high-throughput microscope (GE Healthcare, Buckinghamshire, UK) with 10x objective. Image processing was performed using the IN Cell Investigator (v1.7) software (GE Healthcare, Buckinghamshire, UK). High Content Analysis (HCA) was used for quantification of immunofluorescence images and was performed as described elsewhere [26;39;41;42]. Briefly, two fluorescence images corresponding to DAPI and primary/antibody/Alexa Fluor® 488-secondary were acquired for each field. No fewer than 1,000 cells were acquired. HCA was performed using the IN Cell Investigator (v1.7) software (GE Healthcare, Buckinghamshire, UK). For the analysis, DAPI staining of the nuclei was used to identify nuclear area and number of cells. The nuclei were defined by using top-hat segmentation, specifying a minimum nucleus area of 100 μm^2^. To determine the cellular expression of the analysed protein, the average intensity of pixels in the reference channel (Alexa Fluor® 488) within the specified nuclear region (Object Nuclear Intensity) was measured. Each cell was assigned a nuclear intensity value for the specific protein expression and that value was used to set up a threshold filter, which determined high levels (positive) and low levels (negative) expressing cells. In order to set the filter cut-off, expression in the control cells was measured to define the negative population followed by the analysis of the positive control. As a result, the software classified each cell as either positive or negative for the expression of the analysed protein and generated a percentage of both cell population (positive and negative) per well. The mean of the nuclear intensity was also routinely analysed and equivalent results were obtained.

### IL-8 and IL-6 detection in conditioned medium (CM)

IL-8 detection in CM was performed by western blot analysis. CM was obtained by incubating cells in serum-free medium for 24h. After concentration by centrifugation at 4000 r.p.m. using AgilentSpin Concentrators (Agilent Technologies Inc.,Wilmington, DE, USA), the CM was normalized to cells number, and processed for Western blot analysis.

IL-6 detection was performed by ELISA using the Human Inflammatory Cytokines Multi-Analyte ELISArray Kit (QIAGEN, Hilden, Germany) following manufacturer's instructions. CM was obtained by incubating cells in complete medium for 24h.

### Western Blotting analysis

Cell lysates were obtained by extraction in RIPA modified buffer (20mM Tris-HCl, pH 7.4, 150mM NaCl, 5mM EDTA, 1% NonidetP-40) supplemented with Complete Mini EDTA-free protease Inhibitor Cocktail (Roche, Manheim, Germany), 1mM Na_3_VO_4_ and 1mM PMSF. Protein samples were quantified by Bradford's assay with BIO-RAD Protein Assay (Bio-Rad, Munchen, Germany). Conditioned medium protein samples were obtained as described above. Protein samples were boiled in NuPAGE LDS sample buffer (Invitrogen, Carlsbad, CA, USA) and separated on 4-12% NuPAGE Novex Gel (Invitrogen, Carlsbad, CA, USA) with MES running buffer. Proteins were transferred onto nitrocellulose filters and immunoblotted with the following primary antibodies to: p16^INK4a^ (BD Becton Dickinson, NJ, USA); p21^CIP1^(Santa Cruz, Biotechnology, Inc, Santa Cruz, CA, USA ); p53 (DO-1, Santa Cruz Biotechnolgy Inc, Santa Cruz, CA, USA); β -Actin (Sigma Aldrich, St Louis, Mo, USA); IL-8 (Abcam, Cambridge, UK). The immunoreactive bands were visualized using horseradish peroxidase-conjugated secondary antibodies followed by enhanced chemiluminescence (GE Healthcare, Buckinghamshire, UK).

### Tumour samples and immunohistochemical analysis

Tumour samples are from patients undergoing surgery at Fondazione IRCCS Istituto Nazionale dei Tumori (Milan, Italy). All patients gave their written informed consent, and the study was approved by the independent ethics committee of Fondazione IRCCS Istituto Nazionale dei Tumori.

Stainings were performed on formalin-fixed, paraffin-embedded tissue sections of 2 μm thickness. Antigen retrieval was performed using 1 mM citrate buffer (pH 6) in an autoclave at 95°C for 15 minutes. Incubation with primary antibodies was performed overnight at 4°C for anti-CXCR2 (1:100, BD, Becton Dickinson, NJ, USA) and for 1 hour at room temperature for anti-human Ki-67 Antigen (Mib1) (DakoCytomation, Glostrup, Denmark). Section were then incubated for 30 minutes with biotinylated anti-mouse, developed using 3,3'-diaminobenzidine (DakoCytomation, Glostrup, Denmark) as chromogen, and finally counterstained with hematoxylin.

p16^INK4a^ staining was performed using CINtec Histologic Kit (mtm laboratories AG, Heidelberg, Germany) following manufacturer's instructions.
