# Correction: Oncogenic *RAS*-induced senescence in human primary thyrocytes: molecular effectors and inflammatory secretome involved

**DOI:** 10.18632/oncotarget.28027

**Published:** 2022-10-14

**Authors:** Maria Grazia Vizioli, Joana Santos, Silvana Pilotti, Mara Mazzoni, Maria Chiara Anania, Claudia Miranda, Sonia Pagliardini, Marco A. Pierotti, Jesus Gil, Angela Greco

**Affiliations:** ^1^Molecular Mechanism Unit, Department of Experimental Oncology and Molecular Medicine, Fondazione IRCCS Istituto Nazionale dei Tumori, Milan, Italy; ^2^Cell Proliferation Group, MRC Clinical Sciences Centre, Imperial College London, Hammersmith Campus, London, UK; ^3^Laboratory of Molecular Pathology, Department of Pathology, IRCCS Foundation - Istituto Nazionale dei Tumori, Milan, Italy; ^4^Scientific Directorate, IRCCS Foundation - Istituto Nazionale dei Tumori, Milan, Italy; ^*^Senior co-authors

**This article has been corrected:** Due to errors during figure assembly, an incorrect blot was used for the p53 panel in Figure 3G. Additionally, the actin panel of Figure 1D is an accidental duplicate of the actin panel in Figure 2H. The corrected Figure 3G and 1D are shown below. The authors declare that these corrections do not change the results or conclusions of this paper.

Original article: Oncotarget. 2014; 5:8270–8283. 8270-8283. https://doi.org/10.18632/oncotarget.2013

**Figure 1:**
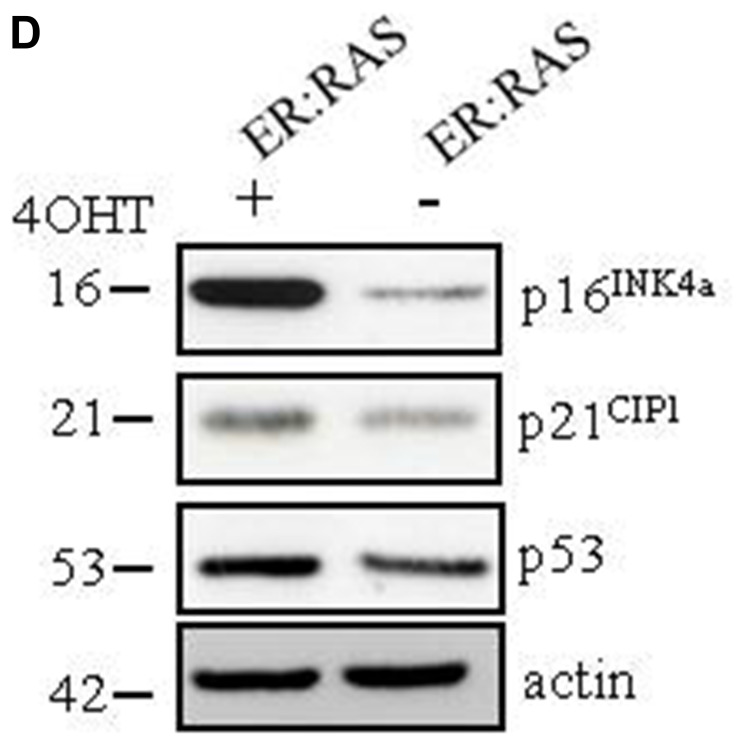
Oncogenic *RAS* triggers senescence in human primary thyrocytes. (**D**) Determination by western blotting of protein levels as indicated in an independent experiment; β-actin serves as loading control. The molecular weight of each protein is indicated. Data are from a representative out of two independent experiments.

**Figure 3:**
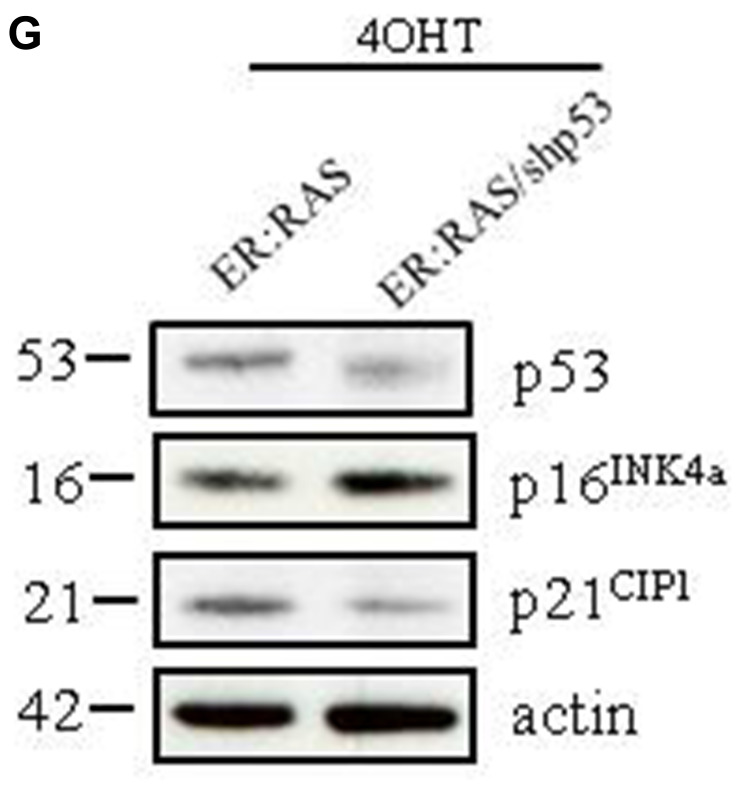
Effect of p53 knockdown on *RAS*-induced senescence in primary thyrocytes. (**G**) Western blotting analysis for the expression of the indicated proteins performed in an independent experiment; β-actin was used as loading control; the molecular weight of each protein is shown. shp53: short hairpin targeting p53; CV: crystal violet ; IF: immunofluorescence.

